# Introduction to the *Toxins*’ Special Issue on Evaluation of Cytotoxicity and Cytoprotection Effects of Natural Toxins

**DOI:** 10.3390/toxins14020114

**Published:** 2022-02-02

**Authors:** Ana Juan-García

**Affiliations:** Laboratory of Food Chemistry and Toxicology, Faculty of Pharmacy, University of Valencia, E-46100 Valencia, Spain; ana.juan@uv.es

The lifestyle associated with good nutritional quality of food is well known for its widely recognized health benefits, especially when rich in bioactive compounds. The classification of bioactive compounds is very wide, including lycopene, carotenoids, and polyphenols (flavonoids and non-flavonoids). Reduced risks of some types of cancer and other diseases have been associated with the adoption of such a diet, as well as having increased antioxidants, inhibitors of lipid peroxidation, a decrease in pro-inflammatory cytokine production, etc.

The presence of natural toxins in food usually happens due to a lack of good harvesting, storage, or packaging practices; climate changes; or atmospheric conditions. Such toxins can have different origins, such as from plants, fungi, algae, bacteria, marine biotoxins including mycotoxins, lectins, furocoumarins, Shiga toxin, ciguatoxins, etc.

The study of mycotoxins has increased in recent years and constitutes a great group to follow in the toxicology field due to their wide and dangerous effects in organs and systems and their presence in many foods, feed, and commodities. Mycotoxins are toxic secondary metabolites produced by filamentous fungi from *Fusarium*, *Alternaria*, and *Penicillium* spp. which spread naturally worldwide. Mycotoxins are also natural contaminants present in food and feed, and several health problems have been evidenced for both humans and animals. All in vitro and in vivo studies are key steps for risk assessment and the following legislation for mycotoxins.

An evaluation of the effects of natural toxins and biologically active compounds of extracts from the plant kingdom constitutes a potential to combat various diseases thanks to its rich content. The focus of this Special Issue of *Toxins* was to gather advances related to the cytotoxicity of natural toxins and the potential for the cytoprotection of natural compounds present in food or plants. In this context, this Special Issue of *Toxins* comprises eight original contributions.

Studies of extracts are presented in this issue from *Artemisia annua, Fridericia chica, Polygonum cuspidatum*, and *Coffea Arabica*, and its protective effects against mycotoxins, such as zearalenone (ZEN), α-zearalenone (α-ZEL), β-zearalenone (β-ZEL), beauvericin (BEA), and patulin (PAT). The following cell lines are used: human neuroblastoma cell line (SH-SY5Y), human embryonic kidney cells (HEK 293T), chicken granulosa cells, bovine aortic ECs (BAECs), bovine mammary epithelial cells (MAC-t), and fibroblast cells (3T3).

The Special Issue starts with the study of Alvarez-Ortega et al. [1] where the protective effects are reported of the hydroethanolic extract (HEFc) from the *Fridericia chica (Bignoniaceae)* leaves grown in the Colombian Caribbean against the cytotoxicity of α-ZEL and β-ZEL on SH-SY5Y cells. A determination of components in extracts through UPLC-QTOF-MS/MS is presented. HEFc showed a significant increase in cell viability after exposure to α-ZEL (25 and 50 µM) and β-ZEL (6–100 µM), and the HEFs are proposed as a valuable source of compounds with antioxidant properties against mycotoxins effects [1].

The Special Issue continues with a study of an extract from *Polygonum cuspidatum* rich in the antioxidant polydatin (PD) [2]. It was assayed in MAC-T cells against ZEN by Fu et al. [2]. It was revealed that ZEA + PD effectively reduced cell oxidative damage compared with the ZEA alone. Furthermore, it was observed that after qPCR analysis on ER stress-related genes and apoptosis genes (*Bax and Bcl*-2), PD down-regulated the expression of these genes as compared with ZEN as well as the caspase-3 activity. It was concluded that PD reduces ZEN-induced apoptosis by inhibiting oxidative damage and ER stress [2].

One of the main beverages drunk all over the world is coffee which is characterized by having a big amount of polyphenols. In addition, the sustainable objective of many companies in upcycling the waste products obtained from coffee is of great concern. Juan-García et al. [3] evaluated coffee extracts from coffee silverskin and spent coffee in a neuroblastoma cell line (SH-SY5Y cells) against beauvericin (BEA) and α-zearalenol (α-ZEL)-induced cytotoxicity with different strategies of treatment (direct, simultaneous, and pre-treatment strategies). Results were very diverse and opposite for some strategies of treatment, concentrations and mycotoxins assayed; however, there is a forthcoming promising use of these unexploited residues in the near future against mycotoxins effects [3].

In an attempt to know the different anti-cancer effects of *Artemisia annua*, extracts using a pressurized cyclic solid-liquid (PCSL) method (phytocomplex extracts) were compared with conventional extraction methods [4]. Effects were tested in the following cancer cell lines from humans, murine, and canines: Balb/c 3T3 mouse cells transformed by simian virus 40 (SVT2), Balb/c 3T3 mouse embryonic fibroblasts (NIH/3T3), human cervical cancer cells (HeLa), and canine osteosarcoma cells (CRL2130). Extracts were not assayed with any natural toxic compounds but evidenced the strong capacity to induce apoptosis and highlighted the possibility of using these extracts as a therapeutical strategy [4].

Studies of mycotoxins in animal cell lines are here presented for two of these cells against ZEN. Two studies in different cell lines are presented: in chicken granulosa cells and bovine aortic ECs (BAECs) [5,6]. The study in chicken granulosa cells was carried out by Zhu et al. [5] and was focused on studying the effect on the function of apoptosis and autophagy by gene expression (Bax, Bcl-2, Cyt C, and Caspase-9 and -3 for apoptosis; and LC3-II and Beclin-1 for autophagy). This cemented an understanding on the signaling pathway for autophagy activated by ZEN [5]. In bovine aortic ECs cells [6], a wide signaling pathway for ZEN was studied in apoptosis through caspase-3 and PARP (poly ADP-ribose polymerase) (ERK1/2/p53/caspase-3), but was independent of ROS production and estrogen receptor activation associated with ZEN.

In the same direction as indicated above, PAT was studied in HEK293 cells in producing and clearing ROS by Liu et al. [7]. This was carried out by studying genes involved in the mitochondrial respiratory chain complex and the role of NAC. It was revealed the importance of PAT in associated health problems [7].

Lastly, in the last chapter of this Special Issue, a validated study for biosafety has been carried out to decrease mycotoxicosis by Huang et al. [8]. NIH/3T3 mouse fibroblasts with products obtained from natural clays and denominated nano-silicate platelets (NSP) were used. ROS, caspase activation, and necroptosis signals have been studied. In conclusion, the study reports that these natural compounds (NPS) might have limited application due to the results obtained [8].
